# Clinically Advanced Pheochromocytomas and Paragangliomas: A Comprehensive Genomic Profiling Study

**DOI:** 10.3390/cancers13133312

**Published:** 2021-07-01

**Authors:** Gennady Bratslavsky, Ethan S. Sokol, Michael Daneshvar, Andrea Necchi, Oleg Shapiro, Joseph Jacob, Nick Liu, Tom S. Sanford, Ruben Pinkhasov, Hanan Goldberg, Jonathan K. Killian, Shakti Ramkissoon, Eric A. Severson, Richard S. P. Huang, Natalie Danziger, Mehdi Mollapour, Jeffrey S. Ross, Karel Pacak

**Affiliations:** 1 Departments of Urology, SUNY Upstate Medical University, Syracuse, NY 13210, USA; michael.daneshvar@nih.gov (M.D.); shapiroo@upstate.edu (O.S.); jacobj@upstate.edu (J.J.); liun@upstate.edu (N.L.); sanforth@upstate.edu (T.S.S.); pinkhasr@upstate.edu (R.P.); goldberh@upstate.edu (H.G.); mollapom@upstate.edu (M.M.); rossj@upstate.edu (J.S.R.); 2 Foundation Medicine, Cambridge, MA 021411, USA; esokol@foundationmedicine.com (E.S.S.); kkillian@foundationmedicine.com (J.K.K.); sramkissoon@foundationmedicine.com (S.R.); eseverson@foundationmedicine.com (E.A.S.); rhuang@foundationmedicine.com (R.S.P.H.); ndanziger@foundationmedicine.com (N.D.); 3 Instituto Nazionale Dei Tumori, 20133 Milan, Italy; andrea.necchi@istitutotumori.mi.it; 4 Section on Medical Neuroendocrinology, Eunice Kennedy Shriver National Institute of Child Health and Human Development, National Institutes of Health, Bethesda, MD 20892, USA; karel.pacak@nih.gov

**Keywords:** pheochromocytoma, paraganglioma, comprehensive genomic profiling, genomic alterations

## Abstract

**Simple Summary:**

Clinically advanced pheochromocytomas and paragangliomas are a rare form of endocrine malignancy which can occur in familial and sporadic clinical settings and feature a variety of genomic alterations. Comprehensive genomic profiling (CGP) was performed to characterize the genomic alterations (GA) in clinically advanced disease to enable the search for potential therapy targets. Although the GA/tumor is relatively low for clinically advanced disease, CGP can reveal important potential targets for therapy in the metastatic setting including *RET*, *NF1* and *FGFR1*. Based on this data, further study of CGP as a method of developing precision therapies for clinically advanced disease appears warranted.

**Abstract:**

Patients with clinically advanced paragangliomas (CA-Para) and pheochromocytomas (CA-Pheo) have limited surgical or systemic treatments. We used comprehensive genomic profiling (CGP) to compare genomic alterations (GA) in CA-Para and CA-Pheo to identify potential therapeutic targets. Eighty-three CA-Para and 45 CA-Pheo underwent hybrid-capture-based CGP using a targeted panel of 324 genes. Tumor mutational burden (TMB) and microsatellite instability (MSI) were determined. The GA/tumor frequencies were low for both tumor types (1.9 GA/tumor for CA-Para, 2.3 GA/tumor for CA-Pheo). The most frequent potentially targetable GA in CA-Para were in *FGFR1* (7%, primarily amplifications), *NF1*, *PTEN*, *NF2*, and *CDK4* (all 2%) and for CA-Pheo in *RET* (9%, primarily fusions), *NF1* (11%) and *FGFR1* (7%). Germline mutations in known cancer predisposition genes were predicted in 13 (30%) of CA-Pheo and 38 (45%) of CA-Para cases, predominantly involving *SDHA/B* genes. Both CA-Para and CA-Para had low median TMB, low PD-L1 expression levels and none had MSI high status. While similar GA frequency is seen in both CA-Para and CA-Para, germline GA were seen more frequently in CA-Para. Low PD-L1 expression levels and no MSI high status argue against strong potential for novel immune checkpoint inhibitors. However, several important potential therapeutic targets in both CA-Para and CA-Para are identified using CGP.

## 1. Introduction

Pheochromocytomas (Pheo) and paragangliomas (Para) are neuroendocrine neoplasms that arise from neural crest derived chromaffin cells located at various sites in the autonomic nervous system and adrenal medulla) [1,2]. While the surgical techniques designed to resect localized Pheo/Para have been greatly customized over time, including the emergence of adrenal cortex sparing sub-total adrenalectomies for Pheo, the improvements in diagnostic capabilities and genetic testing have also caused significant rethinking in uncovering new genetic, biochemical, and other biomarkers suggesting the presence and risk for future development of recurrent and metastatic disease [3,4,5].

Clinically advanced Pheo (CA-Pheo) and Para (CA-Para) are now recognized as being among the more common non-thyroid endocrine malignancies [1,2,4]. With malignancy defined by the development of metastatic lesions either in bones or lymph nodes, the prognosis for CA-Para and CA-Pheo is variable and greatly impacted by the extent and sites of metastases, their biochemical phenotype and underlying mutational landscape, as well as their responsiveness to both local and systemic therapies [6,7]. While some therapies have had success in controlling the progression of these clinically advanced tumors, a significant number of patients are either refractory to their selected therapy at the outset or develop recurrent or widely metastatic disease while on therapy. This led investigators to consider searching for new druggable targets and applications to treat these refractory patients [8].

In the following comprehensive genomic profiling (CGP) study, we compared GA in two separate cohorts of CA-Para and CA-Pheo to learn whether these two different tumor types have similar patterns of mutations and whether CA-Para and CA-Pheo have potential to respond to targeted therapies or immunotherapy.

## 2. Methods

The Western Institutional Review Board (Protocol No. 20152817) issued an approval waiver of informed consent and a HIPAA waiver of authorization for this study. Using either paraffin tissue blocks or a minimum of 40 micron thick unstained tissue section on glass slides, DNA was extracted from clinically advanced cases of 83 CA-Para and 45 CA-Pheo that had progressed to inoperable recurrent disease or clinically evident metastatic disease at the time the sample was submitted for DNA sequencing. Using the medical records and corresponding pathology reports for relevant information, it could be determined that 100% of Pheo/Para samples were obtained either from metastatic site biopsies or from sites of unresectable loco-regional disease. The central laboratory (Foundation Medicine, Cambridge, MA) utilized for the CGP analysis in this study is certified by the Clinical Laboratory Improvement Amendments (CLIA) act and accredited by the CAP (College of American Pathologists). A board-certified anatomic central laboratory pathologist reviewed the medical records and hematoxylin and eosin-stained slides taken from the paraffin block/thick sections sent in by the referring pathology department and assigned the diagnosis of either CA-Para or CA-Pheo at that time prior to DNA extraction. DNA was extracted as previously described [9] only from cases that featured a minimum of 20% tumor nuclei vs benign nuclei and yielded a minimum of 50 ng of extracted DNA from the submitted sample. Cases submitting lacking 20% of tumor nuclei or less than 50 ng of extracted DNA were rejected and not included in this study. After DNA extraction and using microscopic glass shards, a library of the patient’s tumor DNA was cut into short segments of DNA averaging 80–120 base-pairs in length. Adaptor-ligation based hybrid capture was then performed for all coding exons from 287 (version 1) to 324 (version 3) cancer-related genes plus select introns from 19 (version 1) to 28 (version 3) genes frequently rearranged in cancer. The Illumina HiSeq instrument was used for DNA sequencing to a mean exon coverage depth of >500X. The computational pipeline used to analyze sequence patterns utilized Bayesian algorithms to identify base substitution mutations, local assembly to identify short insertions and deletions, comparisons with process matched normal controls to determine gene amplifications and homozygous deletions (copy number changes) and the analysis of chimeric read pairs to identify gene rearrangements and gene fusions [9]. Given the impure nature of clinical samples received in the central laboratory, the test was previously optimized and validated to detect base substitutions at a ≥5% mutant allele frequency (MAF), indels with a ≥10% MAF with ≥99% accuracy, and fusions occurring within baited introns/exons with >99% sensitivity to maximize mutation-detection accuracy (sensitivity and specificity) [9]. The prediction of germline status for this study used the extracted somatic DNA and was performed using a computational approach to distinguish somatic vs. germline origin of genomic alterations from deep sequencing of cancer specimens without a matched normal sample. Using this algorithm, the germline status is successfully predicted in 90–95% of cases with 99.7% accuracy [10]. For clinical patient reports, non-driver germline variants documented in the dbSNP database (dbSNP142; http://www.ncbi.nlm.nih.gov/SNP/, accessed on 20 June 2019) with two or more counts in the ExAC database (http://exac.broadinstitute.org/, accessed on 20 June 2019) or recurrent variants of unknown significance that were predicted by the internally developed algorithm [10] to be germline were not included. However, known driver germline events (e.g., documented hereditary *BRCA1/2* and deleterious *TP53* mutations) were included in patient reports. Using the Catalog of Somatic Mutations in Cancer (COSMIC v62), the known confirmed somatic alterations in this study were listed as biologically significant [11]. For known tumor suppressor genomic alterations, all inactivating events including truncation mutations and deletions were also considered significant and listed in the patient reports and included in this study. Microsatellite instability (MSI) status was determined using the DNA sequencing results across the coding regions of >300 genes. The MSI-High (MSI-H), MSI ambiguous, and MS stable (MSS) categories were assigned by the unsupervised clustering of specimens for which MSI status was previously assessed via gold standard methods (e.g., IHC [11]). Using 0.9 to 1.1 megabases (Mb) of sequenced DNA for each case, the tumor mutational burden (TMB) was determined using the number of somatic base substitution or indel alterations per Mb after filtering to remove known somatic and deleterious mutations as previously described [12]. The DAKO 22C3 antibody was used to determine PD-L1 expression using 5-micron tissue sections. Following the current practice for non-small cell lung carcinomas, only tumor cell membrane staining was scored with 0% defined as negative, low-level staining defined as 1–49% tumor cell expression and high-level staining defined as ≥50% tumor cell expression. 

## 3. Results

The clinical and genomic findings of the 45 CA-Pheo and 83 CA-Para patients are shown in Table 1 which also demonstrates the most common targetable and untargetable GA in each group. The primary tumor was used for sequencing in 27% of the CA-Pheo cases and a metastatic site biopsy or resection in 73%. For CA-Para, the primary tumor was sequenced in 17% of cases and a metastatic site in 83% of cases. The individual known and likely GA, tumor cell proportion and clinical data in the CA-Para and CA-Pheo groups are shown in Supplemental Table S1 (empty columns indicate no copy gain or loss). In addition to the patient’s age and gender and site of sample sequenced, these tables include all classes of GA including short variant base substitutions, short insertions and deletions, copy number changes including amplifications and large deletions and genomic fusions and rearrangements as well as the chromosomal site of the GA, the exact coverage depth, the copy numbers for gene amplifications and the mutant allele frequencies for the short variant mutations.

**Figure 1:**
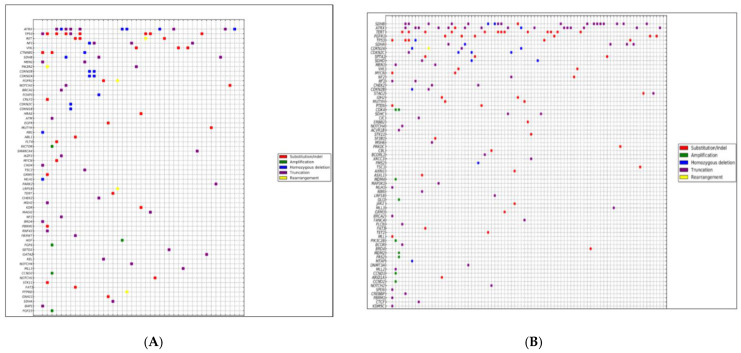
Dot blot charts showing the frequencies of genomic alterations per tumor for the CA-Pheo Figure (**A**) and CA-Para Figure (**B**) patients.

For the CA-Pheo group, the mean GA per tumor was 2.3. The number of GA per tumor varied from 1 GA per tumor seen in nearly three-fourths of the cases to 4 or more GA per tumor with one case featuring 11 GA (Figure 1A). The GA per tumor distribution was similar for the CA-Para patients which featured an average of 1.9 GA per tumor with more than two-thirds of the cases having only 1 GA per tumor. One CA-Para case had 19 GA (Figure 1B).

**Figure 2:**
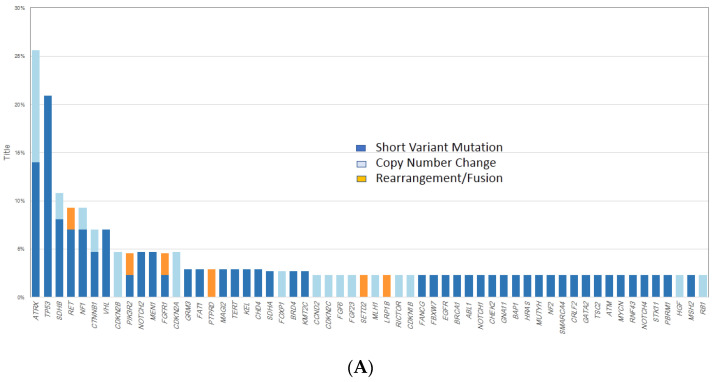

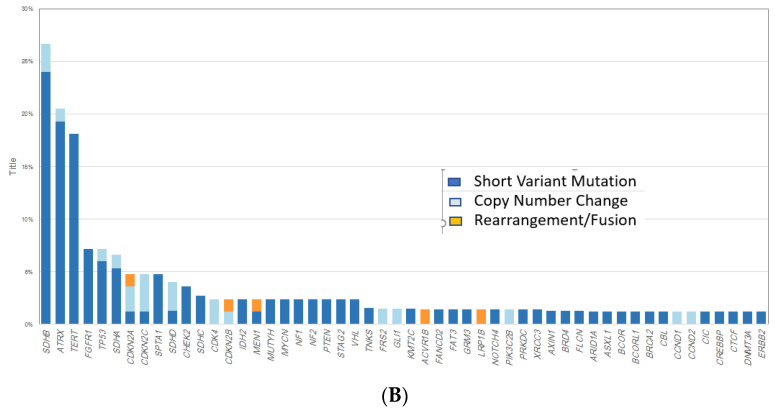
Longtail plots of the frequencies of genomic alterations in CA-Pheo (**A**) and CA-Para (**B**) tumor samples.

Based on existing regulatory approvals and the status of mechanism-driven clinical trials, the most frequent GA considered to be not clinically actionable at the current time in the CA-Pheo group were *ATRX* (24%), *TP53* (20%) *SDHB* (16%), *CTNNB1* (7%), *VHL* (7%) and *CDKN2A/2B*, *PIK3R2*, *NOTCH2,* and *MEN1* all at 4% frequencies (Figure 2A). For the CA-Para group, the most frequently altered not currently actionable genes were *SDHB* (27%), *ATRX* (21%), *TERT* (18%), *TP53* (7%), and *SDHA* (6%) (Figure 2B). The most frequent potentially targetable GA for CA-Pheo were in *RET* (9%, primarily fusions), *NF1* (11%) and *FGFR1* (7%) (Figure 2A). The most frequent potentially targetable GA in CA-Para were in *FGFR1* (7%, primarily amplifications), *NF1*, *PTEN*, *NF2* and *CDK4* (all 2%) (Figure 2B).

**Figure 3:**
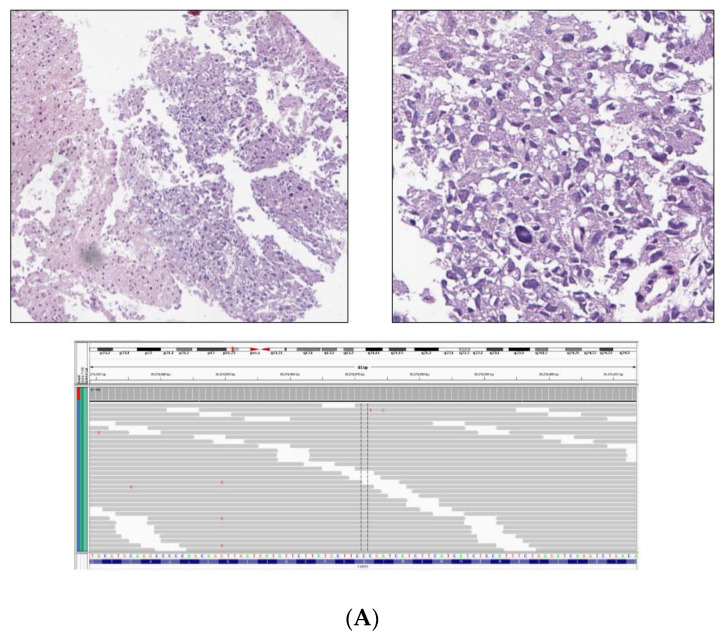

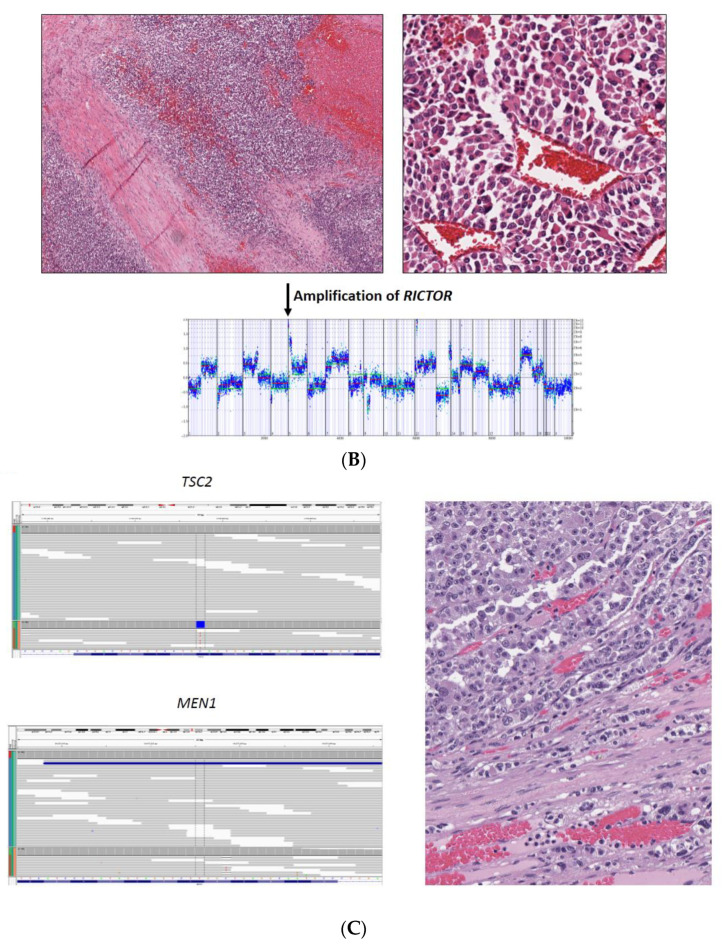

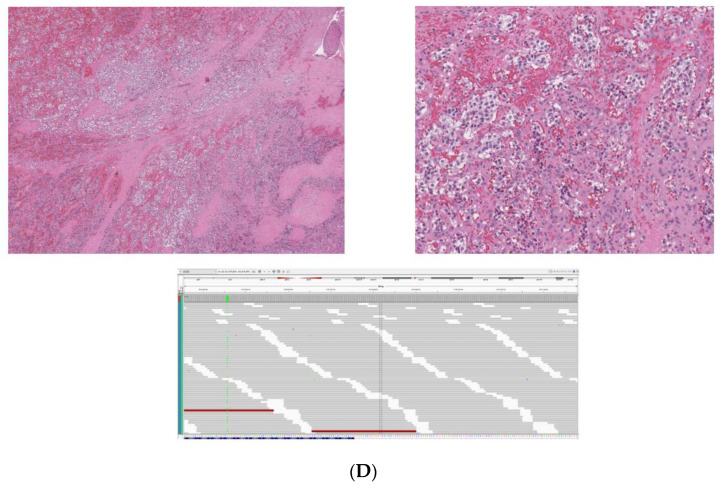
Case examples of genomic alterations associated with CA-Pheo and CA-Para patients. (**A**). *FGFR1* mutation-driven CA-Pheo. Metastatic adrenal Pheo to the liver in a 57-year-old Latino female (case number CA-Pheo45). (**A**) (upper left) shows a low magnification of the metastatic lesion with liver tissue to the left (hematoxylin and eosin X 50). In the higher magnification in (**A**) (upper right) to the upper right, the pleomorphic CA-Pheo is seen with micro focal necrosis (hematoxylin and eosin X 200). In (**A**) (lower center) the only genomic alteration in this sporadic Pheo was an activating mutation in the *FGFR1* gene (N546K) seen in the International Genome Viewer (IGV) plot This CA-Pheo was MSI stable and had a tumor mutation burden of 4 mutations/MB of sequenced DNA. In (**B**). *RICTOR* amplification as a potential target in CA-Pheo (case number CA-Pheo10) is shown. Sporadic clinically advanced Pheo in a 45-year-old Caucasian man. Comprehensive genomic profiling of the primary tumor revealed amplification of the *RICTOR*, *CCND2*, *FGF23* and *FGF6* genes along with short variant mutations in *ATM* L2211fs*4 and *CTNNB1* S45P. (**B**) (upper left) shows a low magnification view of the primary tumor revealing geographic necrosis (hematoxylin and eosin X 50). (**B**) (upper right) shows a higher magnification of the CA-Pheo showing high grade nuclear pleomorphism (hematoxylin and eosin X 200). The copy number plot shown in (**B**) (lower center) highlights the amplification of the *RICTOR* gene which is localized at chromosome 5p 13.1. (**C**) describes a *TSC2* inactivating mutation in a CA-Pheo with germline *MEN1* mutation (case number CA-Pheo42). This case is clinically advanced Pheo of the left adrenal in a 71-year-old African American woman. The primary tumor in (**C**) (right) was 8 cm in largest diameter, featured focal necrosis and lymphovascular invasion (hematoxylin and eosin X 200). This tumor was negative for PD-L1 expression on immunohistochemistry (Dako 22C3 antibody). Comprehensive genomic profiling revealed short variant mutations in *TSC2* Q166* as seen in the IGV plot in (**C**) (upper left). There was also a *CTNNB1* loss exons 2-3 and a predicted to be germline *MEN1* splice site 928-9_942del24 mutation seen in the IGV plot in (**C**) (lower left). (**D**) describes an *NF2* mutation-driven CA-Para (case number CA-Para78) that presented as a recurrent clinically advanced Para of the right ear canal in a 35-year-old Caucasian man that invaded the temporal bone. Comprehensive genomic profiling revealed an *NF2* E463K base substitution mutation and no other genomic alterations. The tumor mutation burden (TMA) was low at 2 mutations per Mb of sequenced DNA and the tumor was MSI Stable. The low magnification image in (**D**) (upper left) shows the CA-Para with fibrous stroma and extensive necrosis (hematoxylin and eosin X 5). The higher magnification image (**D**) (upper right) shows individual cell necrosis, hemorrhage and fibrosis (hematoxylin and eosin X 200). The IGV image in (**D**) (lower center) demonstrates the *NF2* inactivating base substitution.

An example of an *FGFR1* driven CA-Pheo is shown in Figure 3A. In Figure 3B is a case study of a CA-Pheo associated with amplification of the *RICTOR* gene which has been associated with responsiveness to MTOR inhibitors. Figure 3C describes an unusual CA-Pheo that arose in a patient with a likely germline mutation in the *MEN1* gene that also featured a *TSC2* inactivating mutation potentially treatable with mTOR inhibition. For the CA-Para patients, the targetable GA included *FGFR1* (7%, primarily amplifications) and *NF1*, *PTEN*, *NF2* and *CDK4* (all 2%) (Figure 2B). An *NF2* mutation-driven CA-Para originating in the right ear canal in a 35-year-old Caucasian man is shown in Figure 3D. *NF2* driven tumors have been shown to respond to combinations of mTOR inhibitors and MEK inhibitors. 

As seen in Table 1, biomarkers predictive of immune checkpoint inhibitor (ICPI) benefit were not identified in either the CA-Pheo or CA-Para groups. There were no cases associated with high microsatellite instability (MSI-High) and the median TMB was low at 1.7 mutations per Mb for the CA-Pheo tumors and 1.2 mutations per Mb for the CA-Para tumors. *PBRM1* GA linked in some studies to benefit from ICPI in renal cell carcinoma were found in only in 1% of CA-Pheo and 2% of CA-Para. Finally, no cases of CA-Pheo or CA-Para featured *CD74* (*PD-L1*) gene amplification which is associated with ICPI benefit and the PD-L1 immunostaining of tumors cells was less than 10% for all tumors.

While no germline sample was tested from any of the patients in this study, using the published SGZ germline prediction algorithm [9], germline mutations in known cancer predisposition genes could be predicted in 14 (31%) of the CA-Pheo and 38 (45%) of the CA-Para cases. In the CA-Pheo patients, germline mutations were predicted in *SDHB*, *RET*, *VHL*, and *MEN1*. For the CA-Para group, predicted germline tumor predisposition gene mutations predominantly involved the *SDHA/B* genes with other rare germline mutations in *BRD4*, *FLCN*, *TSC1*, *CHEK2*, *TNKS*, *MUTYH*, *FAT3*, *PTEN*, *TNKS*, *IDH2*, *FANCA*, *TET2*, and *VHL*.

## 4. Discussion

This study describes the largest series of clinically CA-Pheos/CA-Paras that was evaluated by comprehensive genomic profiling. Although relatively infrequent when compared to other cancers, genomic alterations identified in Pheo/Para could have significant implications for the planning of treatment in patients whose tumor has either relapsed or progressed to metastatic disease.

Combination chemotherapy using cyclophosphamide, vincristine, and dacarbazine known as the CVD regimen [13], temozolomide, streptozotocin and other chemotherapeutic agents have all been used to treat CA-Pheo/CA-Para patients [14,15]. In addition, in some recurrent or metastatic Pheo/Para, radiofrequency ablation (RFA), ^131^I-MIBG, ^177^Lu-DOTATATE, or external beam radiation have also been used [16,17]. Furthermore, kinase and mTOR pathway inhibitors have also been used to treat both CA-Pheo and CA-Para; nevertheless, this regimen has not employed specific biomarkers or companion diagnostic tests and the results, to date, have been mixed [18]. The overall prognosis for CA-Pheo and CA-Para when they evolve into refractory disease progression resistant to all currently available therapies is usually poor. Thus, there is an unmet need to find new approaches to treat patients with refractory CA-Pheo and CA-Para.

The results shown here revealed that CA-Pheo and CA-Para feature lower frequencies of GAs per sample than routinely seen in more common cancer types [19,20]. For CA-Pheo, the most frequent potentially targetable GA identified in this study were in *RET*, *NF1* and other mTOR pathway genes and *FGFR1*. *RET* alterations included activating base substitutions and gene fusions found in 9% of patients. *RET* GA have been widely studied in CA-Pheo [20]. Broad-based tyrosine kinase inhibitors (TKI) such as cabozantinib are approved for the treatment of *RET* mutations (base substitutions) in medullary thyroid cancers occurring both in sporadic forms and as a component of the MENII syndromes [19]. More recently, *RET* fusions have emerged as significant genomic targets in non-small cell lung cancer [21] and other carcinomas.

While *FGFR1* mutation was reported in only one of 398 Pheo but not in 15 Para analyzed in the COSMIC dataset (August 2018) our study has identified it in 7% and 7% of CA-Pheo and CA-Para, respectively. The greater frequency of *FGFR1* mutation seen in both CA-Pheo and CA-Para seen in the current study may reflect the fact that all the tumors sequenced were clinically advanced, clinically advanced and included many metastases. Tumors with alterations that activate *FGFR1* may be sensitive to FGFR family inhibitors including the approved multikinase inhibitors pazopanib and ponatinib [22]. Recently, a more specific FGFR inhibitor, erdafitinib, has been approved for treatment of urothelial carcinoma of the bladder [23] and is now in development for the treatment of other solid tumors.

*RICTOR* (rapamycin-insensitive companion of mTOR or raptor-independent companion of mTOR) encodes an mTOR-binding protein that is part of the mTORC2 complex [24]. *RICTOR* amplification has been detected in 0.6% of cases in the Pheo and Para TCGA dataset [25] and was similarly identified in only 2% of CA-Pheo in the current study. Tumors with *RICTOR* amplification have shown sensitivity to inhibitors of mTORC225 and everolimus has shown efficacy in *RICTOR* amplified NSCLC [26]. Currently, in addition to the MTOR pathway inhibitor everolimus which is approved for treating breast cancer patients with ER+ disease, additional novel MTOR inhibitors are in early and late-stage clinical trials for the treatment of a wide variety of solid tumors. and may show promise for Pheo/Para patients.

In Figure 3D, CGP revealed an *NF2* E463K base substitution mutation and no other genomic alterations in this CA-Para. This GA is known to disrupt the FERM domain of *NF2* and is inactivating. NF2 mutations have not been previously described in CA-Para patients and there are no *NF2* mutations in the TCGA dataset [24]. Based on the strong clinical evidence from multiple published case reports as well as from preclinical studies, there is considerable evidence that solid tumors that harbor inactivating mutations in *NF2* may be sensitive to treatment strategies that employ MTOR inhibitors such as everolimus as well to the MEK inhibitor drugs such as trametinib and cobimetinib [27].

A variety of biomarkers that have linked to efficacy and resistance to immune checkpoint inhibitor (ICPI) therapies were evaluated in this study. These immunotherapy-related biomarkers included PD-L1 expression determined by IHC and PD-L1 amplification, high MSI, high TMB and BRAF and *PBRM1* mutations detected by CGP. These biomarkers have all been linked to benefit from ICPI treatments in other tumor types including NSCLC, bladder cancer, colorectal cancer and melanoma [28,29], but were only rarely identified in the CA-Pheo and CA-Para sequenced in this study. In addition, biomarkers linked to ICPI resistance and hyper-progression such as *STK11* mutations and *MDM2* amplification were not identified in the CA-Pheo and CA-Para cohorts [30]. In summary, these data indicate that it is unlikely, at least for the foreseeable future, that immunotherapy will play a major role in the treatment strategies for clinically advanced refractory CA-Pheo and CA-Para.

The limitation of this study includes the fact that a formal germline sample obtained from each patient was not separately sequenced to confirm the “predicted” germline status that was generated from using the extracted somatic DNA from the archived clinical tumor sample. This published algorithm is designed to determine germline status of gene sequences from somatic samples and has a high accuracy when used to “predict” the germline status of the alterations that were discovered [10]. The findings of a 30% germline genetic predisposition frequency for the CA-Pheo and 45% frequency for the CA-Para cohorts are similar to previous reports for clinically advanced Pheo/Para patients [10,31].

## 5. Conclusions

In summary, the types and frequencies of clinically actionable and not currently clinically actionable GA in CA-Pheo and CA-Para may have similar opportunities for matching small subsets of patients to a variety of targeted therapies with some of them including kinase and MTOR inhibitors already available on the market. Whenever possible and for patients with metastatic sites safe for biopsy, a post-treatment metastatic site biopsy is preferred over the pre-treatment primary tumor for sequencing as this sample may display GA that reflect resistance mechanisms for the patient’s previous systemic therapies especially when targeted therapies have been utilized. In that regard, the emerging use of next-generation sequencing based liquid biopsies may also be relevant for the CA-Pheo and CA-Para patients as genomic alterations. MSI status and TMB can now be determined with this approach that is reporting a high frequency of successful results capable of guiding therapy decisions. Similar to other cancer types, the results of this study show that the genetic mutational landscape of CA-Pheo and CA-Para is broad, and a search for one “magic drug” is unlikely to be successful, favoring drug or therapeutic modality combination.

## Figures and Tables

**Table 1:** Clinical and Genomic Findings in clinically advanced Pheochromocytomas and Paragangliomas.

1.3 to 2.4 Mutations/Mb	CA-Pheo	CA-Para
Number of Cases	45	83
Age in years (range)	53 (7–78)	48 (10–80)
Males/Females	21/24	50/33
Sample Type Used for Sequencing	Primary Tumor 27% Metastatic Site 73%	Primary tumor 17% Metastatic Site 83%
GA per tumor	2.3	1.9
Predicted Frequency of Germline GA	31%	43%
Most common Untargetable GA	*ATRX* (24%) *TP53* (20%) *SDHB* (16%) *CTNNB1* (7%) *VHL* (7%) *CDKN2A/2B*, *PIK3R2*, *NOTCH2* and *MEN1* (all 4%)	*SDHB* (27%) *ATRX* (21%) *TERT* (18%) *TP53* (7%) *SDHA* (7%)
Most common Targetable GA	*RET* (9%) *NF1* (11%) *FGFR1* (7%)	*FGFR1* (7%) *NF1* (2%) *PTEN* (2%) *NF2* (2%) *CDK4* (2%)
*CD274* amplification	0%	0%
*PBRM1* GA	2%	1%
MSI	0%	0%
Median TMB mut/Mb	1.7	1.2
TMB ≥ 10/20 mut/Mb	2%/0%	6%/2%
PD-L1 Expression low/high	0%/0%	14%/0%

## Data Availability

Data used for this study is made publically available in Supplementary Table S1.

## Supplementary Materials

The following are available online at https://www.mdpi.com/article/10.3390/cancers13133312/s1, Table S1: Clinical and Genomic Findings in CA-Pheo and CA-Para Patients.

## References

[1] Lam A.K.-Y. (2017). Update on Adrenal Tumours in 2017 World Health Organization (WHO) of Endocrine Tumours. Endocr. Pathol..

[2] Williams M.D., Tischler A.S. (2017). Update from the 4th Edition of the World Health Organization Classification of Head and Neck Tumours: Paragangliomas. Head Neck Pathol..

[3] Crona J., Lamarca A., Ghosal S., Welin S., Skogseid B., Pacak K. (2019). Genotype–phenotype correlations in pheochromocytoma and paraganglioma: A systematic review and individual patient meta-analysis. Endocr. Relat. Cancer.

[4] Thompson L.D.R. (2002). Pheochromocytoma of the Adrenal Gland Scaled Score (PASS) to Separate Benign from Malignant Neoplasms. Am. J. Surg. Pathol..

[5] Waingankar N., Bratslavsky G., Jimenez C., Russo P., Kutikov A. (2016). Pheochromocytoma in Urologic Practice. Eur. Urol. Focus.

[6] Guerrero M.A., Schreinemakers J.M., Vriens M.R., Suh I., Hwang J., Shen W.T., Gosnell J., Clark O.H., Duh Q.-Y. (2009). Clinical Spectrum of Pheochromocytoma. J. Am. Coll. Surg..

[7] Lenders J.W.M., Duh Q.-Y., Eisenhofer G., Gimenez-Roqueplo A.-P., Grebe S.K.G., Murad M.H., Naruse M., Pacak K., Young W.F. (2014). Pheochromocytoma and Paraganglioma: An Endocrine Society Clinical Practice Guideline. J. Clin. Endocrinol. Metab..

[8] Björklund P., Pacak K., Crona J. (2016). Precision medicine in pheochromocytoma and paraganglioma: Current and future concepts. J. Intern. Med..

[9] Frampton G.M., Fichtenholtz A., Otto G.A., Wang K., Downing S.R., He J., Schnall-Levin M., White J., Sanford E.M., An P. (2013). Development and validation of a clinical cancer genomic profiling test based on massively parallel DNA sequencing. Nat Biotechnol..

[10] Sun J.X., He Y., Sanford E., Montesion M., Frampton G.M., Vignot S., Soria J.-C., Ross J.S., Miller V.A., Stephens P.J. (2018). A computational approach to distinguish somatic vs. germline origin of genomic alterations from deep sequencing of cancer specimens without a matched normal. PLoS Comput. Biol..

[11] Forbes S.A., Beare D., Gunasekaran P., Leung K., Bindal N., Boutselakis H., Ding M., Bamford S., Cole C., Ward S. (2015). COSMIC: Exploring the world’s knowledge of somatic mutations in human cancer. Nucleic Acids Res..

[12] Chalmers Z.R., Connelly C.F., Fabrizio D., Gay L., Ali S.M., Ennis R., Schrock A., Campbell B., Shlien A., Chmielecki J. (2017). Analysis of 100,000 human cancer genomes reveals the landscape of tumor mutational burden. Genome Med..

[13] Feldman J.M. (1983). Treatment of metastatic pheochromocytoma with streptozocin. Arch. Intern. Med..

[14] Dahia P.L.M. (2014). Pheochromocytoma and paraganglioma pathogenesis: Learning from genetic heterogeneity. Nat. Rev. Cancer.

[15] Fishbein L., Ben-Maimon S., Keefe S., Cengel K., Pryma D.A., Loaiza-Bonilla A., Fraker D.L., Nathanson K.L., Cohen D.L. (2017). SDHB mutation carriers with malignant pheochromocytoma respond better to CVD. Endocr. Relat. Cancer.

[16] Chino J.P., Sampson J.H., Tucci D.L., Brizel D.M., Kirkpatrick J.P. (2009). Paraganglioma of the Head and Neck. Am. J. Clin. Oncol..

[17] Venkatesan A.M., Locklin J., Lai E.W., Adams K.T., Fojo A.T., Pacak K., Wood B.J. (2009). Radiofrequency Ablation of Metastatic Pheochromocytoma. J. Vasc. Interv. Radiol..

[18] Oh D.-Y., Kim T.-W., Park Y.S., Shin S.J., Shin S.H., Song E.-K., Lee H.J., Lee K.-W., Bang Y.-J. (2012). Phase 2 study of everolimus monotherapy in patients with nonfunctioning neuroendocrine tumors or pheochromocytomas/paragangliomas. Cancer.

[19] Watson I., Takahashi K., Futreal P.A., Chin L. (2013). Emerging patterns of somatic mutations in cancer. Nat. Rev. Genet..

[20] Turchini J., Cheung V.K.Y., Tischler A.S., De Krijger R.R., Gill A.J. (2018). Pathology and genetics of phaeochromocytoma and paraganglioma. Histopathology.

[21] Drilon A., Wang L., Arcila M.E., Balasubramanian S., Greenbowe J.R., Ross J.S., Stephens P.J., Lipson D., Miller V.A., Kris M.G. (2015). Broad, Hybrid Capture–Based Next-Generation Sequencing Identifies Actionable Genomic Alterations in Lung Adenocarcinomas Otherwise Negative for Such Alterations by Other Genomic Testing Approaches. Clin. Cancer Res..

[22] Katoh M. (2019). Fibroblast growth factor receptors as treatment targets in clinical oncology. Nat. Rev. Clin. Oncol..

[23] Markham A. (2019). Erdafitinib: First Global Approval. Drugs.

[24] Fishbein L., Leshchiner I., Walter V., Danilova L., Robertson A.G., Johnson A.R., Lichtenberg T.M., Murray B.A., Ghayee H.K., Else T. (2017). Comprehensive Molecular Characterization of Pheochromocytoma and Paraganglioma. Cancer Cell.

[25] Gkountakos A., Pilotto S., Mafficini A., Vicentini C., Simbolo M., Milella M., Giampaolo T., Aldo S., Emilio B., Vincenzo C. (2018). Unmasking the impact of Rictor in cancer: Novel insights of mTORC2 complex. Carcinogenesis.

[26] Sakre N., Wildey G., Behtaj M., Kresak A., Yang M., Fu P., Dowlati A. (2016). RICTOR amplification identifies a subgroup in small cell lung cancer and predicts response to drugs targeting mTOR. Oncotarget.

[27] Tolcher A.W., Bendell J.C., Papadopoulos K.P., Burris H.A., Patnaik A., Jones S.F., Rasco D., Cox D.S., Durante M., Bellew K.M. (2015). A phase IB trial of the oral MEK inhibitor trametinib (GSK1120212) in combination with everolimus in patients with advanced solid tumors. Ann. Oncol..

[28] Chan T., Yarchoan M., Jaffee E., Swanton C., Quezada S., Stenzinger A., Peters S. (2019). Development of tumor mutation burden as an immunotherapy biomarker: Utility for the oncology clinic. Ann. Oncol..

[29] Goodman A.M., Piccioni D., Kato S., Boichard A., Wang H.-Y., Frampton G., Lippman S.M., Connelly C., Fabrizio D., Miller V. (2018). Prevalence of PDL1 Amplification and Preliminary Response to Immune Checkpoint Blockade in Solid Tumors. JAMA Oncol..

[30] Kato S., Goodman A., Walavalkar V., Barkauskas D.A., Sharabi A., Kurzrock R. (2017). Hyperprogressors after Immunotherapy: Analysis of Genomic Alterations Associated with Accelerated Growth Rate. Clin. Cancer Res..

[31] Buffet A., Ben Aim L., Leboulleux S., Drui D., Vezzosi D., Libé R., Ajzenberg C., Bernardeschi D., Cariou B., Chabolle F. (2019). Positive Impact of Genetic Test on the Management and Outcome of Patients with Paraganglioma and/or Pheochromocytoma. J. Clin. Endocrinol. Metab..

